# Age differences in the association of comorbid burden with adverse outcomes in SARS-CoV-2

**DOI:** 10.1186/s12877-021-02340-5

**Published:** 2021-07-06

**Authors:** A. M. O’Hare, K. Berry, V. S. Fan, K. Crothers, M. C. Eastment, J. A. Dominitz, J. A. Shah, P. Green, E. Locke, G. N. Ioannou

**Affiliations:** 1 Division of Nephrology, Veterans Affairs Puget Sound Healthcare System and University of Washington, 1660 South Columbian Way, Seattle, WA 98108 USA; 2 Research and Development, Veterans Affairs Puget Sound Health Care System, Seattle, WA USA; 3 Division of Pulmonary and Critical Care, Veterans Affairs Puget Sound Healthcare System and University of Washington, Seattle, WA USA; 4 Division of Allergy and Infectious Disease, Veterans Affairs Puget Sound Healthcare System and University of Washington, Seattle, WA USA; 5 Division of Gastroenterology, Veterans Affairs Puget Sound Healthcare System and University of Washington, Seattle, WA USA

## Abstract

**Background:**

Older age and comorbid burden are both associated with adverse outcomes in SARS-CoV-2, but it is not known whether the association between comorbid burden and adverse outcomes differs in older and younger adults.

**Objective:**

To compare the relationship between comorbid burden and adverse outcomes in adults with SARS-CoV-2 of different ages (18–64, 65–79 and ≥ 80 years).

**Design, setting, and participants:**

Observational longitudinal cohort study of 170,528 patients who tested positive for SARS-CoV-2 in the US Department of Veterans Affairs (VA) Health Care System between 2/28/20 and 12/31/2020 who were followed through 01/31/2021.

**Measurements:**

Charlson Comorbidity Index (CCI); Incidence of hospitalization, intensive care unit (ICU) admission, mechanical ventilation, and death within 30 days of a positive SARS-CoV-2 test.

**Results:**

The cumulative 30-day incidence of death was 0.8% in cohort members < 65 years, 7.1% in those aged 65–79 years and 20.6% in those aged ≥80 years. The respective 30-day incidences of hospitalization were 8.2, 21.7 and 29.5%, of ICU admission were 2.7, 8.6, and 11% and of mechanical ventilation were 1, 3.9 and 3.2%. Median CCI (interquartile range) ranged from 0.0 (0.0, 2.0) in the youngest, to 4 (2.0, 7.0) in the oldest age group. The adjusted association of CCI with all outcomes was attenuated at older ages such that the threshold level of CCI above which the risk for each outcome exceeded the reference group (1st quartile) was lower in younger than in older cohort members (*p* < 0.001 for all age group interactions).

**Limitations:**

The CCI is calculated based on diagnostic codes, which may not provide an accurate assessment of comorbid burden.

**Conclusions:**

Age differences in the distribution and prognostic significance of overall comorbid burden could inform clinical management, vaccination prioritization and population health during the pandemic and argue for more work to understand the role of age and comorbidity in shaping the care of hospitalized patients with SARS-CoV-2.

**Supplementary Information:**

The online version contains supplementary material available at 10.1186/s12877-021-02340-5.

## Introduction

Older age is the strongest risk factor for infection with the severe acute respiratory virus syndrome coronavirus-2 (SARS-CoV-2) and older adults have been especially hard-hit during the pandemic [1, 2]. Older adults with SARS-CoV-2 are at increased risk for hospitalization [3–5], critical illness [4, 6–13], prolonged hospitalization [11, 14] and mortality [8, 9, 14–29] compared with their younger counterparts. Because a disproportionate number of hospitalizations and deaths in patients with coronavirus disease 2019 (COVID-19) occur among older adults [30–32], the burden of SARS-CoV-2 has been greatest in countries [33–36] and communities [37] with older populations, and in healthcare facilities [38–40] and health systems serving older adults [41].

However, the prevalence of most comorbid conditions increases with age, which can make it challenging to disentangle the separate effects of age and comorbid burden on outcomes among patients infected with SARS-CoV-2. Prior studies have reported an association between Charlson comorbidity score and other measures of comorbid burden with adverse outcomes in SARS-CoV-2 after adjustment for age [41–49] and differences across age groups in clinical presentation and outcomes of SARS-CoV-2 [11, 14, 27, 31, 50–53]. There are also reports of age differences in the association of individual comorbid conditions [54–56] with adverse outcomes in infected patients.

While few studies have examined the relationship between age, comorbid burden and adverse outcomes in SARS-CoV-2, available data suggest that the relationship between these factors may be complex. Among the first 11,122 infected patients in Denmark, mortality rates were noted to be extremely low in those < 80 years old without comorbid conditions but uniformly high in those ≥80 years old regardless of the number of comorbid conditions [57]. Because both age and comorbidity have figured prominently in guidelines for clinical care, vaccination and social distancing during the pandemic, a more detailed understanding of the relationship of age and comorbid burden with a range of adverse outcomes among adults infected with SARS-CoV-2 could inform clinical care, current and future vaccine prioritization and prognostication [58, 59] and efforts to estimate disease burden and service needs related to SARS-CoV-2 [33].

The US Department of Veterans Affairs (VA) supports the largest integrated national health care system in the US and provides care for more than six million veterans annually [60]. Because the VA serves both younger and older adults and many veterans have multiple comorbid conditions [61], the system may offer unique insights into the relationship between age, comorbid burden and adverse outcomes in those infected with SARS-CoV-2. We designed a study to evaluate differences across age groups in the association of comorbid burden with a range of adverse outcomes among patients infected with SARS-CoV-2.

## Methods

### Data source and study population

The VA uses a single comprehensive electronic healthcare information network. Data elements from patients’ electronic medical records at individual VA facilities are stored centrally in the VA’s Corporate Data Warehouse (CDW) which is maintained by the VA Informatics and Computing Infrastructure (VINCI). To support research on the health system impacts of the SARS-CoV-2 pandemic, VA maintains the VA National Surveillance Tool, which includes updated clinical and administrative data extracts for all patients tested for SARS-CoV-2 within the VA, with adjudication of all positive test results [60]. Using this resource, we assembled a cohort of all Veterans who tested positive for SARS-CoV-2 nucleic acid by polymerase chain reaction (PCR) at least once between February 28, 2020 and December 31, 2020 with complete information on test date (*n* = 170,528). The index date for the present study was defined as the date of each patient’s earliest positive test result unless this occurred within the first 15 days of a hospital admission, in which case we used the date of hospital admission as the index date. Follow-up for all study outcomes was available through January 31, 2021, allowing for analysis of all outcomes occurring within 30 days after the index date for all cohort members. This study was approved by the Institutional Review Board of the Veterans Affairs Puget Sound Healthcare System which granted a waiver of informed consent as the study was deemed minimal risk because it involved secondary analyses of existing data and could not otherwise have been conducted due to the large size of the cohort and inclusion deceased patients. Our research was conducted in accordance with the Declaration of Helsinki.

### Exposure

The primary exposure was each patient’s Charlson Comorbidity Index (CCI) score (categorized by approximate quartile of the distribution within our cohort as 0, 1, 2–3 or ≥ 4) based on International Classification of Diseases 10th Revision (ICD-10) codes recorded in VA administrative data on or within 2 years before the index data [62].

### Outcomes

The following outcomes were ascertained for the 30-day period following the index data using the VA National Surveillance Tool [60]: 1) hospitalization; 2) ICU admission; 3) mechanical ventilation; and 4) death.

### Covariates

All analyses were stratified by patients’ age group on the index date (categorized as 18–64, 65–79 and ≥ 80 years) and adjusted for sex, race (Black, White, Other), Hispanic ethnicity, body mass index body (BMI) (categorized as underweight (< 18.5 kg/m^2^), normal weight (18.5–24.9 kg/m^2^) overweight (25–29.9 kg/m^2^), stage 1 obesity (30–34.9 kg/m^2^) and stages 2 & 3 obesity (≥35 kg/m^2^)) and U.S. Federal Region. To account for potential age differences in outcomes within each age group, multivariate analyses were also adjusted for age as a continuous variable.

### Statistical analysis

We used Pearson’s χ2 test to compare patient characteristics across age groups. We plotted 30-day cumulative rates of mortality, hospitalization, ICU admission, and mechanical ventilation by age using a lowess smoother with a bandwidth of 80%. We used a time-to-event analysis accounting for censoring at the time of death to calculate the crude cumulative 30-day incidence of hospitalization, ICU admission, mechanical ventilation, and death within each age group.

We used Cox proportional hazards models stratified by age group to measure the adjusted association of CCI with hospitalization, ICU admission, mechanical ventilation, and death within the first 30 days after the index date after adjustment for age (as a continuous variable), sex, race, Hispanic ethnicity, BMI, and region of residence. We used a two-sided *p*-value threshold of < 0.05 to assess statistical significance. We confirmed there were no violations of the proportional hazard assumption via visual inspection of log-log graphs**.** All analyses were stratified by VA medical center. A likelihood ratio test (or χ^2^ test of predicted hazard ratios) was used to evaluate for interaction between age group and CCI in adjusted analyses.

We conducted a supplementary analysis in which we used competing risk models to estimate the 30-day cumulative incidence of hospitalization, ICU admission and mechanical ventilation and to measure the adjusted association of CCI with each of these non-death outcomes [63]. We also repeated the primary analyses using approximate quartiles of the Elixhauser Comorbidity Index (ECI).

All analyses were conducted using Stata/MP Version 15 (StataCorp, LLC. College Station, TX).

## Results

### Baseline characteristics

Among the 170,528 patients with a positive test for SARS-CoV-2 during the ascertainment period, 99,483 were aged 18–64, 55,013 were aged 65–79, and 26,032 were aged ≥80 years (Table 1). From the youngest to the oldest age group, there were decreases in the percentage of women, patients of Black and Other race, patients of Hispanic ethnicity and overweight patients. The median CCI (interquartile range) ranged from 0.0 (0.0, 2.0) in the youngest to 4 (2.0, 7.0) in the oldest age group. More than half (54.6%) of patients in the youngest age group had a CCI of 0 as compared with 8.8% of those in the oldest age group, while more than half (57.5%) of those in the oldest age group had a CCI ≥ 4 as compared with 10.7% of those in the youngest age group.

**Table 1:** Baseline characteristics of patients with a positive test for SARS-CoV-2 in the VA healthcare system, by age group

Characteristic	18-64 years ***N***=99,483	65-79 years ***N***=55,013	≥80 years ***N***=26,032	All patients ***N***=170,528	***P*** value
**Sex**					< 0.001
Women, %	26.2	3.8	2.2	16.7	
Men, %	73.8	96.2	97.8	83.3	
**Race**					< 0.001
White, %	51.7	72.5	77.2	60.8	
Black, %	23.4	18.3	12.8	20.8	
Other, %	3.3	2.1	1.7	2.7	
Missing/Unknown	21.5	7.1	8.3	15.6	
**Ethnicity**					< 0.001
Non-Hispanic	69.4	89.6	89.7	77.8	
Hispanic	11.9	6.3	5.3	9.5	
Missing/Unknown	18.7	4.2	5	12.7	
**Body mass index (kg/m**^**2**^**)**					< 0.001
<18.5 (underweight), %	0.4	1.2	2.7	0.9	
18.5-24.9 (normal weight), %	9.9	16	33.4	14.1	
25.29.9 (overweight), %	25.7	34.2	37.2	29.5	
30-34.9 (Obese I), %	26.2	27.6	17.1	25.8	
≥35 (Obese II and III), %	23.4	19.4	6.2	20.5	
Missing, %	14.5	1.6	3.4	9.3	
**Charlson Comorbidity Index (median (IQR))**	0.0 (0.0,2.0)	3.0 (2.0,6.0)	4.0 (2.0,7.0)	1.0 (0.0,4.0)	< 0.001
**Charlson Comorbidity Index (%)**					< 0.001
0	54.6	11.2	8.8	36.3	
1	18.3	12	9.5	15.5	
2-3	16.4	27.5	24.2	20.7	
≥4	10.7	49.3	57.5	27.6	
**Median (IQR)**	**18-64 years**	**65-79 years**	**≥80 years**	**All patients**	
Hospital LOS	5 (3-9)	7 (4-14)	9 (5-15)	7 (4-13)	
ICU (LOS)	4 (2-8)	5 (3-10)	5 (2-9)	5 (3-9)	

### Crude 30-day cumulative incidence of hospital admission, ICU admission, mechanical ventilation, and death by age group

**Fig. 1:**
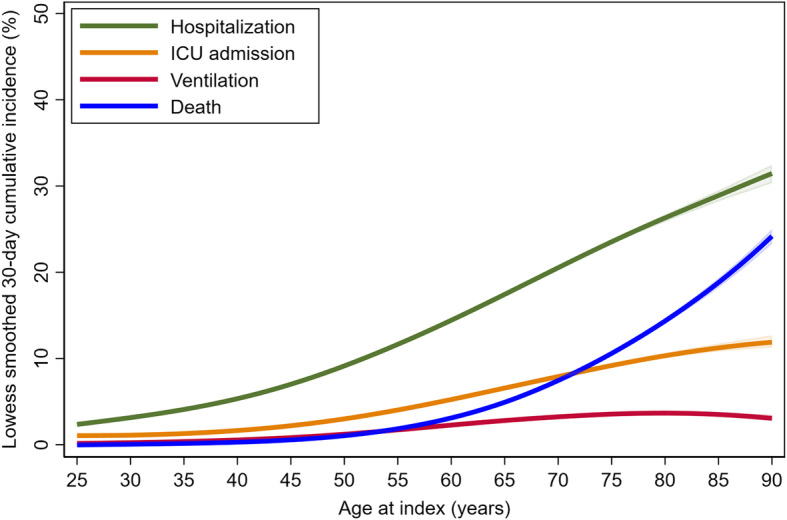
Lowess smoothed 30-day cumulative rates of hospitalization, ICU admission, mechanical ventilation and death by age at baseline

**Fig. 2:**
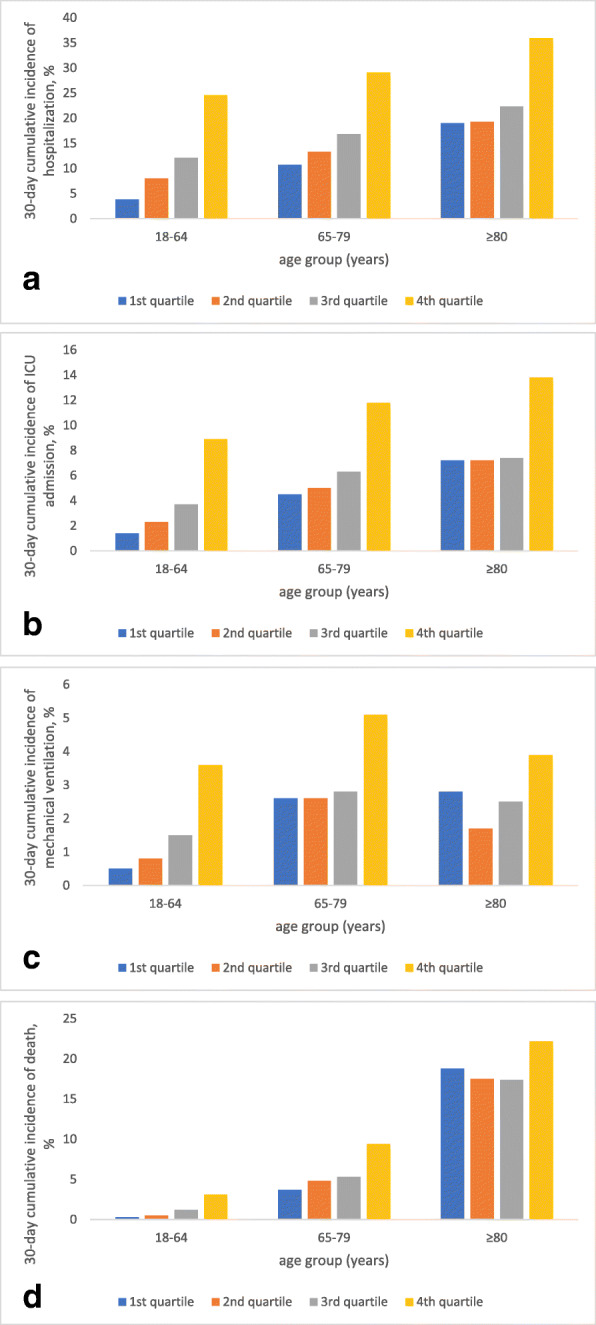
30-day cumulative incidence of hospitalization (**a**), ICU admission (**b**), mechanical ventilation (**c**) and death (**d**) by age group

The cumulative incidence of death increased exponentially with increasing age while the incidence of hospitalization, ICU admission, and mechanical ventilation plateaued at older ages, with rates of mechanical ventilation declining beyond the age of 80 (Fig. 1). The 30-day incidence of death increased across age groups and quartiles of CCI for all outcomes except for mechanical ventilation (Fig. 2). In all age groups, the incidence of hospitalization (Fig. 2a), ICU admission (Fig. 2b) and mechanical ventilation (Fig. 2c) were extremely high for those with a CCI in the fourth quartile. The incidence of death increased linearly with CCI quartile for those aged < 65 and 65–79 years but was extremely high in all quartiles of CCI for those aged ≥80 years (Fig. 2d). Death rates were higher for those aged ≥80 years than for any other age group regardless of CCI. The 30-day incidence of death for those aged< 65 years with a CCI in the 4th quartile was similar to that for patients aged 65–79 years with a CCI in the first quartile. Median hospital length of stay ranged from 5 (interquartile range (IQR) 3–9) in the youngest age group to 9 (IQR 5–15) in the oldest age group and median ICU length of stay was 4 (IQR 2–8) in those < 65 years, 5(IQR 3–10) for those aged 65–79 and 5 (IQR 2–9) for those aged ≥80 years.

### Adjusted analyses of the association of CCI with hospitalization, ICU admission, mechanical ventilation and death after stratification by age group

The adjusted association of CCI with all outcomes varied systematically across age groups and was attenuated at older ages (*p* values for all age group interactions < 0.001) (Table 2). Among patients < 65 years, those with a CCI in the 2nd (vs. 1st) quartile (and higher) were at increased risk for hospitalization (adjusted hazard ratio (aHR) 1.43, 95% confidence interval (CI) 1.33, 1.53) and ICU admission (aHR 1.25, 95% CI 1.10, 1.42) and those with a CCI in the 3rd (vs. 1st) quartile (and higher) were at increased risk for mechanical ventilation (aHR 1.41, 95% CI 1.16, 1.70) and death (aHR 1.74, 95% CI 1.38, 2.20). Among those aged 65–79 years, risk of hospitalization was increased for those with a CCI in the 2nd (vs. 1st) quartile of CCI (aHR 1.19, 95% CI 1.07–1.31), risk of ICU admission was increased for those with a CCI in the 3rd (vs. 1st) quartile (aHR 1.33, 95% CI 1.16–1.53), risk of mechanical ventilation was increased for those with a CCI in the 4th (vs.1st) quartile (aHR 1.69, 95% CI 1.43–2.01) and risk of death was increased for those with a CCI in the 2nd (vs. 1st) quartile (aHR 1.34, 95% CI 1.12–1.60). However, among patients ≥80 years, only those with CCI in the 4th (vs.1st) quartile of CCI were at increased risk for hospitalization (aHR 1.55, 95% CI 1.36, 1.78), ICU admission (aHR 1.51, 95% CI 1.21, 1.89) and death (aHR 1.41, 95% CI 1.22, 1.64). Risk for mechanical ventilation did not vary substantially across quartiles of CCI in this oldest age group and was lowest for those with a CCI in the 2nd quartile (aHR 0.55, 95% CI 0.33, 0.93 (Table 2).

**Table 2:** Adjusted association of Charlson Comorbidity Index with hospitalization, ICU admission, mechanical ventilation and death, stratified by age group

	18-64 years ***N***=99,483	65-79 years ***N***=55,013	≥80 years ***N***=26,032	All patients ***N***=170,528	
	Adjusted^ab^ hazard ratio (95% confidence intervals)	Adjusted^ab^ hazard ratio (95% confidence intervals)	Adjusted^ab^ hazard ratio (95% confidence intervals)	Adjusted^ab^ hazard ratio (95% confidence intervals)	P for interaction with age group
**Hospitalization**					
Number of hospitalizations	8,110	11,907	4,676	24,693	
30-day cumulative incidence of hospitalization (per 100 patients)	8.2	21.7	29.5	14.5	< 0.001***
Median length of stay (IQR), days	4 (2-8)	6 (3-13)	8 (4-15)	6 (3-12)	
Charlson Comorbidity Index					< 0.001***
0	1	1	1	1	
1	1.40 (1.31-1.51)***	1.19 (1.07-1.31)**	0.91 (0.77-1.08)	1.37 (1.29-1.44)***	
2-3	1.86 (1.73-1.99)***	1.48 (1.36-1.62)***	1.01 (0.88-1.17)	1.75 (1.66-1.84)***	
≥4	3.35 (3.13-3.58)***	2.51 (2.31-2.73)***	1.55 (1.36-1.78)***	2.91 (2.77-3.05)***	
**ICU admission**					
Median Length of stay (IQR), days	4 (1-7)	4 (2-10)	4 (2-8)	4 (2-9)	
Number of ICU admissions	2,705	4,709	1,712	9,126	< 0.001***
30-day cumulative incidence of ICU admission (per 100 patients)	2.7	8.6	11	5.4	
Charlson Comorbidity Index					< 0.001***
0	1	1	1	1	
1	1.25 (1.10-1.42)***	1.11 (0.94-1.30)	0.92 (0.70-1.23)	1.25 (1.14-1.37)***	
2-3	1.70 (1.51-1.92)***	1.33 (1.16-1.53)***	0.91 (0.71-1.16)	1.60 (1.47-1.74)***	
≥4	3.43 (3.05-3.85)***	2.36 (2.08-2.69)***	1.51 (1.21-1.89)***	2.88 (2.66-3.12)***	
**Mechanical ventilation**					< 0.001***
Number receiving mechanical ventilation	1,017	2,109	490	3,616	
30-day incidence of mechanical ventilation (per 100 patients)	1	3.9	3.2	2.1	
Charlson Comorbidity Index					< 0.001***
0	1	1	1	1	
1	0.91 (0.73-1.13)	0.97 (0.78-1.20)	0.55 (0.33-0.93)*	1.06 (0.92-1.23)	
2-3	1.41 (1.16-1.70)***	1.00 (0.83-1.21)	0.76 (0.51-1.13)	1.44 (1.27-1.64)***	
≥4	2.72 (2.26-3.26)***	1.69 (1.43-2.01)***	1.03 (0.71-1.48)	2.43 (2.16-2.74)***	
**Death**					< 0.001***
Number of deaths	770	3,897	3,255	7,922	
30-day cumulative incidence of death (per 100 patients)	0.8	7.1	20.3	4.6	
Charlson Comorbidity Index					< 0.001***
0	1	1	1	1	
1	0.98 (0.75-1.29)	1.34 (1.12-1.60)**	1.05 (0.88-1.26)	1.29 (1.15-1.44)***	
2-3	1.74 (1.38-2.20)***	1.45(1.25-1.70)***	1.08 (0.92-1.27)	1.53 (1.38-1.69)***	
≥4	3.26 (2.61-4.08)***	2.48 (2.14-2.86)***	1.41 (1.22-1.64)***	2.35 (2.14-2.59)***	

^a^ Adjusted for Federal Emergency Management Agency region: 1 (Connecticut, Massachusetts, Maine, New Hampshire, Rhode Island, Vermont), 2 (New Jersey, New York, Puerto Rico), 3 (District of Columbia, Delaware, Maryland, Pennsylvania, Virginia, West Virginia), 4 (Alabama, Florida, Georgia, Kentucky, Mississippi, North Carolina, South Carolina, Tennessee), 5 (Illinois, Indiana, Michigan, Minnesota, Ohio, Wisconsin), 6 (Arkansas, Louisiana, New Mexico, Oklahoma, Texas), 7 (Iowa, Kansas, Missouri, Nebraska), 8 (Colorado, Montana, North Dakota, South Dakota, Utah, Wyoming), 9 (Arizona, California, Guam, Hawaii, Nevada), 10 (Alaska, Idaho, Oregon, Washington) and all characteristics listed in Table 1 with the exception that age was modeled as a continuous variable.

^b^Stratified by station.

*P<0.05, **P<0.01, ***P<0.001

Results for non-death outcomes were similar when we used a competing risk model (Additional file: Table 1). The results of a sensitivity analysis examining associations of ECI with study outcomes after stratification by age were similar to the primary analysis (Additional file: Table 2).

## Discussion

Among VA patients who tested positive for SARS-CoV-2 during the first 10 months of the pandemic, there were marked differences across age groups in the prevalence and prognostic significance of comorbid burden. Associations of comorbid burden with adverse outcomes were generally attenuated at older ages leading to systematic differences across age groups in the threshold level of the CCI (and ECI) associated with an increased risk for adverse outcomes. In cohort members < 65 years and 65–79 years, risk for most outcomes was increased for patients with a CCI in the second or third quartile (CCI of 1 or 2–3, respectively) or higher. On the other hand, among those aged ≥80 years, only a CCI in the 4th quartile (CCI ≥4) was associated with an increased risk for hospitalization, ICU admission and death and risk of mechanical ventilation did not vary greatly with CCI.

The disproportionate number of deaths occurring among older adults is a distinguishing feature of the SARS-CoV-2 pandemic as compared with prior influenza pandemics [32]. The very steep age gradient in risks of death and hospitalization and strong association of comorbid burden with adverse outcomes among younger and middle aged members of our cohort generally supports the US Centers for Disease Control-recommended approach to vaccine prioritization based on age and the presence of comorbid conditions among younger and middle-aged (i.e. < 65 years) adults [64]. However, as we and others have argued elsewhere, there may be opportunities to refine ongoing vaccine prioritization strategies by accounting for the presence of sizeable differences within age groups in risk for adverse outcomes [65–67].

Consistent with the crude mortality rates reported by Reilev et al. for the first 11,122 infected patients in Denmark, risk of death was extremely high for the oldest members of our cohort regardless of CCI [57]. However, there were substantial differences in the relative risk for all adverse outcomes across quartiles of comorbid burden in younger age groups, including those aged 65–79 years. Further, rates of hospitalization, ICU admission and mechanical ventilation for patients with a CCI in the fourth quartile were extremely high regardless of age. Prioritization for vaccination and risk stratification based on comorbid burden may be especially impactful for those aged 65–79 years given that their absolute mortality and hospitalization rates substantially exceed those aged < 65 years. Collectively, these findings suggest there may be opportunities to refine ongoing vaccine allocation strategies to improve risk stratification within this relatively large high-risk group.

While prior studies have described strong associations of CCI and other measures of comorbid burden with adverse outcomes in patients infected with SARS-CoV-2 after adjusting for age [41–48], none to our knowledge have evaluated the adjusted association of comorbid burden with adverse outcomes after stratification by age group. However, our major finding of systematic differences across age groups in the strength and magnitude of the association of comorbid burden with hospitalization, death and ICU admission among patients with SARS-CoV-2 seems consistent with other work describing age differences in the association of individual comorbid conditions [54–56] with adverse outcomes among infected patients. An attenuation of the association of comorbid burden with adverse outcomes in patients with SARS-CoV-2 at older ages also resonates with earlier work demonstrating that in older adults, the presence or absence of particular comorbid conditions and/or abnormalities in specific disease markers often have less prognostic significance than more global measures of functional status and frailty that are not necessarily tied to the presence or severity of specific underlying disease processes [68–70]. Our findings may also suggest the need for alternative approaches to risk stratification within the oldest age group, perhaps based on non-disease-specific measures such as frailty and functional status (71) or different threshold levels of comorbid burden.

Consistent with earlier studies conducted in a range of different populations infected with SARS-CoV-2 [8, 9, 14–29, 71], the age gradient for crude mortality was extremely steep and mortality rates increased exponentially while slopes for non-death outcomes plateaued at older ages. Age-related increases in rates of ICU admission and mechanical ventilation among members of our cohort were much less steep than those for mortality and hospitalization. Of particular note, crude rates of mechanical ventilation peaked among patients in their late 70’s and declined at older ages and the association of CCI with mechanical ventilation was substantially attenuated among those aged 65–79 years and extinguished in the oldest age group. These findings support the possibility that clinical management of hospitalized patients infected with SARS-CoV-2 might vary depending on the patient’s age and comorbid burden [72]. Collectively, our work argues for studies to understand the impact of age and comorbidity on real-world care practices for patients infected with SARS-CoV-2.

Limitations of our study include first, that our results may not be generalizable to other populations, particularly non-veteran populations and those that include a higher percentage of women. Second, ascertainment of comorbidity was limited to information included in ICD-10 codes which may not fully capture the severity of individual comorbid conditions or comorbid disease burden. Third, we only had information on hospitalizations occurring within the VA or under VA Community Care, thus our results may underestimate rates of non-death outcomes among cohort members. Fourth, due to selective testing for SARS-CoV-2 within our health system, our results may not be generalizable to infected Veterans not tested within the VA and may be subject to bias based on age and comorbidity. Finally, our analyses are adjusted for a limited number of measured patient characteristics and may reflect confounding by unmeasured characteristics.

In conclusion, while prior studies have demonstrated strong associations between older age and comorbid burden with the severe manifestations of COVID-19, none have rigorously evaluated for age differences in the strength and magnitude of the association of comorbid burden with a range of adverse outcomes. Our finding of systematic differences across age groups in the distribution and prognostic significance of comorbid burden among patients infected with SARS-CoV-2 could help to inform clinical care, ongoing vaccine prioritization and prognostication. Age differences in outcomes for hospitalized patients with SARS-CoV-2 also argue for more work to understand the role of age and comorbid burden in shaping clinical care and decision-making.

## Supplementary Information

**Additional file 1.**

## Data Availability

Our data were obtained under data use agreement and cannot be shared beyond the study team members authorized to access the data. The data sources we used are accessible to other VA researchers for approved projects: https://www.hsrd.research.va.gov/for_researchers/cyber_seminars/archives/video_archive.cfm?SessionID=3834
