# Supplementary material for: Age differences in the association of comorbid burden with adverse outcomes in SARS-CoV-2
Source: BMC Geriatr. 2021 Jul 6;21:415. doi: 10.1186/s12877-021-02340-5 (PMC8258273; doi:10.1186/s12877-021-02340-5)
Supplement: Supplementary file 1 — Additional file 1. [file 12877_2021_2340_MOESM1_ESM.docx]

**Appendix**

**Appendix Table 1: Adjusted association of Charlson Comorbidity Index with hospitalization stratified by age group with death treated as a competing risk**

|  | **18-64 years**  **N=99,483** | **65-79 years**  **N=55,013** | **≥80 years**  **N=26,032** | **All patients**  **N=170,528** |  |
| --- | --- | --- | --- | --- | --- |
|  | Adjusted*† hazard ratio (95% confidence intervals) | Adjusted*† hazard ratio (95% confidence intervals) | Adjusted*† hazard ratio (95% confidence intervals) | Adjusted‡† hazard ratio (95% confidence intervals) | P for interaction with age group |
| **Hospitalization** |  |  |  |  |  |
| Number of hospitalizations | 8,110 | 11,907 | 4,676 | 24,693 |  |
| 30-Day cumulative incidence of hospitalization (per 100 patients) | 8.2 | 21.6 | 29.2 | 14.5 | < 0.001*** |
| Charlson Comorbidity Index |  |  |  |  |  |
| 0 | 1 | 1 | 1 | 1 |  |
| 1 | 1.42 (1.33-1.52)*** | 1.20 (1.09-1.32)*** | 0.90 (0.77-1.06) | 1.39 (1.32-1.46)*** |  |
| 2-3 | 1.89 (1.77-2.02)*** | 1.50 (1.38-1.63)*** | 1.03 (0.90-1.18) | 1.79 (1.70-1.87)*** |  |
| ≥4 | 3.53 (3.31-3.77)*** | 2.60 (2.41-2.82)*** | 1.67 (1.47-1.89)*** | 3.06 (2.92-3.21)*** |  |
| **ICU admission** |  |  |  |  |  |
| Number of ICU admissions | 2,705 | 4,709 | 1,712 | 9,126 |  |
| 30-Day cumulative incidence of ICU admissions (per 100 patients) | 2.7 | 8.6 | 10.7 | 5.4 | < 0.001*** |
| Charlson Comorbidity Index |  |  |  |  |  |
| 0 | 1 | 1 | 1 | 1 |  |
| 1 | 1.18 (1.05-1.33)** | 1.11 (0.94-1.30) | 0.87 (0.67-1.15) | 1.20 (1.10-1.32)*** |  |
| 2-3 | 1.64 (1.47-1.83)*** | 1.37 (1.19-1.57)*** | 0.91 (0.72-1.14) | 1.58 (1.46-1.71)*** |  |
| ≥4 | 3.51 (3.15-3.92)*** | 2.49 (2.19-2.83)*** | 1.62 (1.32-2.01)*** | 2.98 (2.76-3.22)*** |  |
| **Mechanical ventilation** |  |  |  |  |  |
| Number of patients who received mechanical ventilation | 1,017 | 2,109 | 490 | 3,616 |  |
| 30-Day cumulative incidence of mechanical ventilation (per 100 patients) | 1 | 3.8 | 3.1 | 2.1 | < 0.001*** |
| Charlson Comorbidity Index |  |  |  |  |  |
| 0 | 1 | 1 | 1 | 1 |  |
| 1 | 0.97 (0.79-1.19) | 0.97 (0.79-1.20) | 0.56 (0.34-0.92)* | 1.11 (0.96-1.27) |  |
| 2-3 | 1.47 (1.23-1.76)*** | 1.03 (0.86-1.23) | 0.80 (0.54-1.17) | 1.51 (1.33-1.71)*** |  |
| ≥4 | 2.94 (2.47-3.50)*** | 1.76 (1.50-2.08)*** | 1.14 (0.81-1.60) | 2.62 (2.32-2.96)*** |  |

‡ Adjusted for Federal Emergency Management Agency region: 1 (Connecticut, Massachusetts, Maine, New Hampshire, Rhode Island, Vermont), 2 (New Jersey, New York, Puerto Rico), 3 (District of Columbia, Delaware, Maryland, Pennsylvania, Virginia, West Virginia), 4 (Alabama, Florida, Georgia, Kentucky, Mississippi, North Carolina, South Carolina, Tennessee), 5 (Illinois, Indiana, Michigan, Minnesota, Ohio, Wisconsin), 6 (Arkansas, Louisiana, New Mexico, Oklahoma, Texas), 7 (Iowa, Kansas, Missouri, Nebraska), 8 (Colorado, Montana, North Dakota, South Dakota, Utah, Wyoming), 9 (Arizona, California, Guam, Hawaii, Nevada), 10 (Alaska, Idaho, Oregon, Washington) and all characteristics listed in Table 1 with the exception that age was modeled as a continuous variable.

† Stratified by station.

*P<0.05, **P<0.01, ***P<0.001

**Appendix Table 2: Adjusted association of Elixhauser Comorbidity Index with hospitalization, ICU admission, mechanical ventilation and death, stratified by age group**

|  | **18-64 years**  **N=99,483** | **65-79 years**  **N=55,013** | **≥80 years**  **N=26,032** | **All patients**  **N=170,528** |  |
| --- | --- | --- | --- | --- | --- |
|  | Adjusted‡† hazard ratio (95% confidence intervals) | Adjusted‡† hazard ratio (95% confidence intervals) | Adjusted‡† hazard ratio (95% confidence intervals) | Adjusted‡† hazard ratio (95% confidence intervals) | P for interaction with age group |
| **Hospitalization** |  |  |  |  |  |
| Number of hospitalizations | 8,110 | 11,907 | 4,676 | 24,693 |  |
| 30-day cumulative incidence of hospitalization (per 100 patients) | 8.2 | 21.7 | 29.5 | 14.5 | < 0.001*** |
| Elixhauser Comorbidity Index |  |  |  |  | < 0.001*** |
| 0 | 1 | 1 | 1 | 1 |  |
| 1-6 | 1.39 (1.30-1.48)*** | 1.32 (1.25-1.40)*** | 1.10 (1.00-1.21) | 1.36 (1.31-1.42)*** |  |
| 7-16 | 1.88 (1.77-2.00)*** | 1.53 (1.45-1.62)*** | 1.21 (1.11-1.32)*** | 1.64 (1.58-1.70)*** |  |
| ≥17 | 3.91 (3.66-4.18)*** | 2.76 (2.63-2.91)*** | 1.98 (1.83-2.15)*** | 2.92 (2.82-3.03)*** |  |
| **ICU admission** |  |  |  |  |  |
| Number of ICU admissions | 2,705 | 4,709 | 1,712 | 9,126 |  |
| 30-day cumulative incidence of ICU admission (per 100 patients) | 2.7 | 8.6 | 11 | 5.4 | < 0.001*** |
| Elixhauser Comorbidity Index |  |  |  |  | < 0.001*** |
| 0 | 1 | 1 | 1 | 1 |  |
| 1-6 | 1.29 (1.15-1.45)*** | 1.36 (1.24-1.49)*** | 1.07 (0.91-1.25) | 1.36 (1.28-1.46)*** |  |
| 7-16 | 1.79 (1.60-2.01)*** | 1.42 (1.30-1.54)*** | 1.15 (1.00-1.33) | 1.55 (1.46-1.65)*** |  |
| ≥17 | 4.34 (3.89-4.84)*** | 2.69 (2.48-2.91)*** | 1.78 (1.56-2.03)*** | 2.90 (2.74-3.07)*** |  |
| **Mechanical ventilation** |  |  |  |  |  |
| Number receiving mechanical ventilation | 1,017 | 2,109 | 490 | 3,616 |  |
| 30-day incidence of mechanical ventilation (per 100 patients) | 1 | 3.9 | 3.2 | 2.1 | < 0.001*** |
| Elixhauser Comorbidity Index |  |  |  |  | < 0.001*** |
| 0 | 1 | 1 | 1 | 1 |  |
| 1-6 | 1.21 (1.01-1.45)* | 1.20 (1.05-1.37)** | 0.96 (0.72-1.27) | 1.29 (1.16-1.42)*** |  |
| 7-16 | 1.71 (1.43-2.05)*** | 1.26 (1.11-1.43)*** | 1.01 (0.78-1.30) | 1.48 (1.35-1.63)*** |  |
| ≥17 | 3.82 (3.20-4.55)*** | 2.24 (2.00-2.51)*** | 1.41 (1.11-1.79)** | 2.59 (2.36-2.84)*** |  |
| **Death** |  |  |  |  |  |
| Number of deaths | 770 | 3,897 | 3,255 | 7,922 |  |
| 30-day cumulative incidence of death (per 100 patients) | 1 | 7 | 20 | 5 | < 0.001*** |
| Elixhauser Comorbidity Index |  |  |  |  | < 0.001 |
| 0 | 1 | 1 | 1 | 1 |  |
| 1-6 | 1.36 (1.11-1.67)** | 1.43 (1.30-1.57)*** | 1.08 (0.98-1.20) | 1.34 (1.26-1.43)*** |  |
| 7-16 | 1.69 (1.37-2.07)*** | 1.28 (1.17-1.41)*** | 1.03 (0.93-1.13) | 1.27 (1.19-1.35)*** |  |
| ≥17 | 3.65 (2.99-4.46)*** | 2.20 (2.02-2.40)*** | 1.23 (1.12-1.36)*** | 1.88 (1.77-2.00)*** |  |

‡ Adjusted for Federal Emergency Management Agency region: 1 (Connecticut, Massachusetts, Maine, New Hampshire, Rhode Island, Vermont), 2 (New Jersey, New York, Puerto Rico), 3 (District of Columbia, Delaware, Maryland, Pennsylvania, Virginia, West Virginia), 4 (Alabama, Florida, Georgia, Kentucky, Mississippi, North Carolina, South Carolina, Tennessee), 5 (Illinois, Indiana, Michigan, Minnesota, Ohio, Wisconsin), 6 (Arkansas, Louisiana, New Mexico, Oklahoma, Texas), 7 (Iowa, Kansas, Missouri, Nebraska), 8 (Colorado, Montana, North Dakota, South Dakota, Utah, Wyoming), 9 (Arizona, California, Guam, Hawaii, Nevada), 10 (Alaska, Idaho, Oregon, Washington) and all characteristics listed in Table 1 with the exception that age was modeled as a continuous variable.

† Stratified by station.

*P<0.05, **P<0.01, ***P<0.001
